# Enrichment of Aerobic and Anaerobic Hydrocarbon-Degrading Bacteria from Multicontaminated Marine Sediment in Mar Piccolo Site (Taranto, Italy)

**DOI:** 10.3390/microorganisms11112782

**Published:** 2023-11-16

**Authors:** Bruna Matturro, Maria Letizia Di Franca, Barbara Tonanzi, Carolina Cruz Viggi, Federico Aulenta, Magda Di Leo, Santina Giandomenico, Simona Rossetti

**Affiliations:** 1 Water Research Institute (IRSA), National Research Council (CNR), 00010 Montelibretti, Italyfederico.aulenta@irsa.cnr.it (F.A.); simona.rossetti@irsa.cnr.it (S.R.); 2 National Biodiversity Future Center, 90133 Palermo, Italy

**Keywords:** polychlorinated biphenyl, polycyclic aromatic hydrocarbons, hydrocarbon-degrading bacteria, reductive dechlorination, *Rhodococcus cerastii*, *Marinobacter salinus*, *Dehalococcoides mccartyi*, multi-contamination, marine sediment

## Abstract

Marine sediments act as a sink for the accumulation of various organic contaminants such as polychlorobiphenyls (PCBs). These contaminants affect the composition and activity of microbial communities, particularly favoring those capable of thriving from their biodegradation and biotransformation under favorable conditions. Hence, contaminated environments represent a valuable biological resource for the exploration and cultivation of microorganisms with bioremediation potential. In this study, we successfully cultivated microbial consortia with the capacity for PCB removal under both aerobic and anaerobic conditions. The source of these consortia was a multicontaminated marine sediment collected from the Mar Piccolo (Taranto, Italy), one of Europe’s most heavily polluted sites. High-throughput sequencing was employed to investigate the dynamics of the bacterial community of the marine sediment sample, revealing distinct and divergent selection patterns depending on the imposed reductive or oxidative conditions. The aerobic incubation resulted in the rapid selection of bacteria specialized in oxidative pathways for hydrocarbon transformation, leading to the isolation of *Marinobacter salinus* and *Rhodococcus cerastii* species, also known for their involvement in aerobic polycyclic aromatic hydrocarbons (PAHs) transformation. On the other hand, anaerobic incubation facilitated the selection of dechlorinating species, including *Dehalococcoides mccartyi*, involved in PCB reduction. This study significantly contributes to our understanding of the diversity, dynamics, and adaptation of the bacterial community in the hydrocarbon-contaminated marine sediment from one sampling point of the Mar Piccolo basin, particularly in response to stressful conditions. Furthermore, the establishment of consortia with biodegradation and biotransformation capabilities represents a substantial advancement in addressing the challenge of restoring polluted sites, including marine sediments, thus contributing to expanding the toolkit for effective bioremediation strategies.

## 1. Introduction

In marine ecosystems, the presence of hydrocarbon pollutants is an increasingly pressing issue, with compounds like polychlorinated biphenyls (PCBs) posing significant ecological and health risks [1,2]. PCBs, classified as aromatic hydrocarbons, have garnered attention due to their toxicity, persisting in marine sediments long after regulation of their production and use in many countries [3,4,5]. Comprising 209 different congeners, PCBs exhibit varying degrees of chlorination on the biphenyl ring, influencing their physical and chemical properties [6]. These compounds not only threaten aquatic ecosystems, but also pose health concerns for human populations residing in coastal regions.

Despite their challenging degradability, specialized microorganisms have evolved the capacity to biodegrade or biotransform PCBs through anaerobic and aerobic pathways [7]. Under anaerobic conditions, the microbial reductive dechlorination of PCBs occurs via multiple pathways, leading to the gradual removal of chlorine atoms and the transformation of highly chlorinated PCBs into less chlorinated forms. These resulting low-chlorinated byproducts exhibit reduced toxicity and bioaccumulation potential, and are more amenable to subsequent aerobic biodegradation [8,9,10].

The anaerobic dechlorination of PCBs within marine sediment environments represents a complex and dynamic process of paramount environmental significance. Under anaerobic conditions, PCB reductive dechlorination unfolds through an intricate network of at least nine distinct biological pathways, denoted as P, H, H’, N, M, Q, T, Z, and LP [11]. This transformative process results in the gradual removal of chlorine atoms from PCB molecules, ultimately yielding less chlorinated congeners. Intriguingly, the microorganisms driving these reductive dechlorination pathways remain largely elusive due to their resistance to laboratory cultivation, contributing to the enigmatic nature of this biodegradative phenomenon. Previous studies have unveiled novel dechlorinating species and their associated enzymes named PCBases, referring to the reductive dehalogenase specifically engaged in the PCB reductive dechlorination process [12]. The genes coding for these enzymes have also been found in PCB-contaminated marine sediments [13,14]. Specialized microorganisms have garnered substantial attention for their pivotal roles in PCB reductive dechlorination processes, contributing to our understanding of the intricate mechanisms underlying PCB biotransformation. Among them, members of the *Chloroflexi* phylum have emerged as prominent players. Notably, *Dehalococcoides mccartyi*, a well-documented dechlorinating organism, has also been associated with PCB reductive dechlorination [12,14,15,16,17,18]. Additionally, *Dehalobium chlorocoercia* DF-1 species have demonstrated their significance in mediating PCB transformation processes [16,19,20].

The aerobic biodegradation of PCBs involves their oxidation, possibly leading to the eventual mineralization of these compounds. Various bacterial species, including *Pseudomonas*, *Alcaligenes, Burkholderia, Acinetobacter, Comamonas, Corynebacterium, Rhodococcus,* and others, are capable of PCB oxidation [7,21,22,23,24,25,26]. Furthermore, it is noteworthy that the majority of these bacterial strains harbor the biphenyl dioxygenase gene (*bphA*), which encodes biphenyl dioxygenase, initiating the catabolic pathway associated with PCB degradation [27]. The *bphA* gene encompasses a substantial Fe–S subunit that bestows substrate specificity, targeting various PCB congeners, as well as other organic pollutants, including polycyclic aromatic hydrocarbons (PAHs), xylene, ethylbenzene, chlorobenzenes, and toluene [28].

Despite the environmental concerns posed by PCBs in marine sediments, autochthonous microbial communities have demonstrated theiradaptability to contaminations over time, developing enhanced biodegradative capabilities compared to uncontaminated environments. Marine sediments exposed to multiple contaminants represent rich sources of microbial diversity, potentially capable of cooperatively metabolizing a broad spectrum of hydrocarbons, although the full extent of the diversity and role of uncultivable microbes in PCB degradation remains unclear. Hence, there is still a need for research to identify the key biological players involved in these processes, with the goal of implementing effective bioremediation strategies either by enhancing microbial consortia or by isolating novel species from the native communities within the impacted environment. Indeed, native microorganisms from contaminated sites represent a valuable resource for assessing a site’s bioremediation potential under controlled conditions, but also for facilitating the exploration of undiscovered metabolic features, providing insights into site biodiversity and biodegradative capabilities, and enabling the identification of potential bioresources (e.g., consortia for bioaugmentation) for broader field-scale applications. Traditionally, culture-dependent techniques have been employed for isolating microorganisms from various environments. Nevertheless, these methods offered only a limited perspective on the microbial community structure within environmental samples. The literature indicates that culture-based approaches can capture less than 1% of microorganisms in diverse environments. This limitation is largely attributed to the labor-intensive nature of these methods, often resulting in an incomplete representation of the characteristics of culturable communities. Consequently, a significant portion of microbial diversity remains poorly explored, forming a vast and uncharacterized biological resource known as the “microbial dark matter”. Understanding this complex realm remains a challenge in current research.

In response to these challenges, advanced biomolecular tools (BMTs) and high-throughput sequencing techniques, based on a culture-independent approach, also known as –omics tools (OTs), have emerged as essential in recent decades. These tools significantly enhance our capacity to investigate microbial communities, functional genes, metabolic pathways, and biomarkers involved in bioremediation processes with unprecedented precision and efficiency. In particular, metagenomic approaches, such as 16S amplicon and shotgun sequencing, have become the primary OTs for gaining comprehensive knowledge of microbial communities. These methods provide a straightforward way to profile microbial communities through the culture-independent analysis of microbial DNA directly extracted from environmental samples. They offer insights into the taxonomic and functional composition of microbial communities across various microbial groups and from a wide range of environmental contexts. However, the effectiveness of sequencing data from environmental samples depends on various factors, such as community complexity, biomass, sequencing technology, and reference databases; these factors can sometimes result in the underrepresentation of certain taxa, especially those with low abundance. The combination of sequencing methods and microbial cultivation is crucial in uncovering the biodiversity and discovering novel microbial species/consortia within a contaminated site.

In this study, we established aerobic and anaerobic microbial consortia using multicontaminated marine sediment collected from the highly polluted basin Mar Piccolo in Taranto, Italy. This site is characterized by elevated levels of persistent organic pollutants, including PCBs, PAHs, dioxins, and heavy metals. Several studies have focused on characterizing the microbiome of marine sediment collected from various sampling points at the site [14,18,29,30]. These studies have also assessed the metabolic potential of the indigenous microbial communities [31,32]. These studies consistently reveal highly specialized microbiomes involved in the degradation of both aromatic and aliphatic hydrocarbons, primarily composed of microorganisms from the *Betaproteobacteriales, Deltaproteobacteria,* and *Chloroflexi* bacterial groups. Based on the previous studies conducted within the same basin, the current research aimed to uncover the bacterial diversity of contaminated marine sediment collected from a single sampling point in the Mar Piccolo of Taranto. Furthermore, we aimed to evaluate the growth dynamics under specific conditions, ultimately developing specialized microbial consortia for both aerobic and anaerobic PCB degradation. In detail, the objectives of this study were to elucidate how anaerobic and aerobic conditions shape microbial community functionality and structure, potentially through the selective enrichment of specific microbial groups. In this study, we used traditional cultivation techniques, coupled with the –omic approach, offering valuable insights into the metabolic potential and environmental adaptations of microorganisms, as well as interspecies synergistic interactions. Furthermore, this study aim at the establishment of consortia with biodegradation and biotransformation capabilities, derived from diverse environmental matrices contaminated with multiple agents. This approach will expand the toolkit available for effective bioremediation strategies, contributing to the comprehensive restoration of contaminated environments, including marine sediments.

## 2. Materials and Methods

### 2.1. Sampling of the Marine Sediment

The marine sediment was collected in June 2016 from the First Inlet of Mar Piccolo in Taranto (coordinates: 40°29′2.42″ N, 17°13′49.76″ E) using a Van Veen grab at a depth of approximately 3.8 m. Three sediment samples were collected by a scuba diver in an area of approximately 1 m^2^. The samples were combined and homogenized to obtain a composite average sample. Following the sampling process, a portion of the sediment was placed in an HDPE (high-density polyethylene) container and stored at −18 °C for subsequent chemical and biomolecular analyses. The sediment sample was chosen from among those sampled within an area characterized in the Life4marpiccolo project (LIFE14/ENV/IT/000461) for testing, on a pilot scale, an innovative technology based on tangential microfiltration, aimed at the in situ removal of only the fine fraction of sediment (i.e., the most contaminated) without making changes to or damaging the marine ecosystem. This sediment was characterized by high concentrations of organic and inorganic contaminants such as metals and PCBs, which represent the most important pollutants in the First Inlet of Mar Piccolo basin in Taranto [33,34].

### 2.2. Microbial Enrichment Cultivation

The multicontaminated marine sediment served as the inoculum for cultivating aerobic and anaerobic consortia, enabling the selection of microorganisms involved in either reductive or oxidative hydrocarbon degradation. Various incubation conditions were explored, including:

(i) Aerobic biostimulation with biphenyl to initiate PCB aerobic transformation (*Ae*);
(ii) Anaerobic incubation without the addition of external electron donors or biostimulating compounds (*Ana*);
(iii) Anaerobic incubation with periodic spikes of PCE (0.8 mM) as an electron acceptor and H_2_ (5 mL) as an electron donor, to stimulate PCB reductive dechlorination (Ana*), and to facilitate the growth of dechlorinating bacteria and enhance the reductive PCB dechlorination, as previously documented [12].

All microbial consortia were established using 50 g of dry marine sediment and 70 mL of synthetic marine water composed of the following components: NaCl 22 g, MgCl_2_·6H_2_O 9.7 g/L, Na_2_SO_4_ 3.7 g/L, CaCl_2_ 1 g/L, KCl 0.65 g/L, NaHCO_3_ 0.2 g/L, and H_3_BO_3_ 0.023 g/L. Specifically, for anaerobic cultures, we prepared sterile serum bottles with a total volume of 160 mL, including 60 mL of headspace. These bottles were sealed with Teflon-faced butyl rubber stoppers and flushed with a N_2_/CO_2_ mixture for 20 min.

For the aerobic consortium (*Ae*), we combined the marine sediment and synthetic marine water in a sterile aerobic flask with a total volume of 160 mL, including 60 mL of headspace. The flask was sealed with a silicone cap and placed on a magnetic plate for stirring to facilitate oxygen transfer. The addition of biphenyl (200 mg/L) to the aerobic culture served as an inducer for PCB aerobic transformation, which is a valuable approach in understanding the potential for aerobic PCB degradation in marine sediments [35].

Additionally, isolation attempts were made from the *Ae* culture by sampling the sediment after 40 and 60 days of aerobic incubation, during which 16S rRNA gene amplicon sequencing revealed strong bacterial selection (see below). Isolates were obtained on agar plates using the ONR7a mineral salts medium as described by Dyksterhouse et al. [36], with biphenyl as the sole carbon source. All cultures were maintained at 20 °C and monitored for 250 days.

### 2.3. DNA Extraction

DNA was extracted from the original marine sediment and from the microbial consortia at various incubation time points (0.5 g of dry sediment per sample). Sampling for the *Ae* culture occurred at 20, 40, 60, 90, 200, and 250 days of incubation. The *Ana* culture was sampled after 20, 60, 90, and 250 days of incubation, while the Ana* culture was sampled at 60, 90, and 250 days of incubation. DNA extractions were carried out using the DNeasy PowerSoil Kit (Qiagen, Italy), following the manufacturer’s instructions. The extracted DNA was eluted in 100 μL of sterile Milli-Q water and stored at −20 °C for subsequent biomolecular analysis.

### 2.4. 16S rRNA Gene Amplicon Sequencing

We used 4 ng of DNA as a template for PCR amplification targeting the V1–V3 region of the 16S rRNA gene. For library construction, the PCR reactions were conducted in a total volume of 25 μL, comprising Phusion Master Mix High Fidelity (Thermo Fisher Scientific, United States) and a final concentration of 0.5 μM for the library adaptors along with 27F and 534R primers. All PCR reactions were performed in duplicate and subsequently pooled. The amplicon libraries were purified using Agencourt^®^ AMpureXP-beads (Beckman Coulter, Brea, CA, USA), and their concentrations were measured using a Qubit 3.0 fluorometer (Thermo Fisher Scientific, Waltham, MA, USA). The purified libraries were equimolarly pooled and then diluted to 4 nM. A Phix control was added at a 10% ratio to the pooled libraries.

Subsequently, the samples underwent paired-end sequencing (2 × 301 bp) on a MiSeq instrument (Illumina, San Diego, CA, USA) using a MiSeq Reagent kit v3, 600 cycles (Illumina, USA), following standard guidelines. The sequencing yielded a total of 3,926,447 raw reads. Bioinformatic analysis was performed after checking read quality with FastQC software (v 0.11.7). Sequences were processed, quality-filtered and analyzed using QIIME2 version 2018.2 [37]. The reads were demultiplexed using the demux plugin (https://github.com/qiime2/q2-demux; accessed on 25 May 2022) and the primer sequences were removed using the cutadapt plugin (https://github.com/qiime2/q2-cutadapt; accessed on 22 February 2022). The DADA2 algorithm was used to denoise paired end sequences, dereplicate them and filter chimeras via the “consensus” method, and resolve amplicon sequence variants (ASVs) [38]. The Silva 132–99 database was used to assign the taxonomy (release December 2017, https://www.arb-silva.de/documentation/release-132/; accessed on 25 May 2022) [39]. A dataset of amplicon sequence variants (ASVs) was generated, and the Shannon index (H) was calculated using PAST 4.0. Principal component analysis (PCA), correspondence analysis and heatmap analysis were conducted with the Clustvis tool [40]. Sequencing data have been deposited in the DDBJ/ENA/GenBank under the BioProject PRJNA843975.

### 2.5. Biomarker Genes Quantification

To quantify biomarker genes involved in the aerobic and anaerobic biodegradation or bio-transformation of PCBs, real-time PCR (qPCR) was employed.

The *bphA* gene was quantified in the *Ae* culture, while the reductive dehalogenase genes (*tceA, bvcA, vcrA*, *pcbA1, pcbA4, pcbA5* genes) were quantified in the *Ana* and Ana* enrichment cultures.

qPCR reactions to quantify *bphA*, *pcbA1, pcbA4* and *pcbA5* genes were conducted in a 20 μL total volume of SsoAdvanced^®^ Universal SYBR^®^ Green Supermix (Biorad, Italy), which included 3 μL of DNA as a template and 300 nM of each primer. Primer sets have been reported in previous studies [14,41].

Furthermore, the quantification of *tceA, bvcA,* and *vcrA* genes in the *Ana* and Ana* consortia was carried out using qPCR reactions with TaqMan^®^ chemistry. A 20 μL total volume reaction was prepared, using the SsoAdvancedTM Universal Probes Supermix (Biorad, Italy), with 3 μL of DNA as a template, 300 nM of each primer, and 300 nM of TaqMan^®^ probes [10]. All qPCR reactions were performed in triplicate using the CFX96 TouchTM Real-Time PCR Detection System (Biorad, Italy). The resulting quantitative data were expressed as gene copy numbers per gram of dry sediment, and error bars were calculated using Microsoft Excel^®^ based on triplicate reactions for each sample.

### 2.6. PCBs and Chlorinated Ethenes Determination

Quantification of PCBs in the sediment was performed by GC–MS upon microwave-assisted solid/liquid extraction. Duplicate extractions were carried out on each sample to ensure reproducibility. In brief, upon sampling the sediment was air-dried at room temperature for 48 h over a hexane-rinsed aluminum foil and then finely ground in an agate mortar. The extraction was performed using a microwave-assisted extraction system (Ethos X, Milestone, Italy). Briefly, 5 g dried samples were accurately weighed into a 100 mL disposable glass vial and then added with 25 mL of a 1:1 acetone/hexane mixture. The extraction was performed for 15 min at 110 °C and 6–10 bars; then, extracts were cooled to room temperature and passed through Na_2_SO_4_ cartridges. Finally, extracts were concentrated by evaporation under a gentle nitrogen stream to a final volume of 5 mL. The extract (1 μL) was injected (in pulsed split-less mode) into a GC–MS (Perkin Elmer Clarus 680/600; column: HP-5 MS (Agilent) 30 m, ID 0.25 mm, 0.25 mm film thickness; carrier gas: helium 1 mL/min; injection temperature: 310 °C; interface temperature: 280 °C; oven temperature program: 70 °C for 2.0 min, then 25 °C/min to 150 °C, then 3 °C/min to 220 °C, then 10 °C/min to 300 °C, hold for 5 min). The Selected Ion Monitoring MS method used for PCBs determination is reported in Table 1. The quantification of PCBs was performed by means of external standards (BP-WD, Native PCB Window Defining Solution/Mixture, Wellington Laboratories). The molar concentrations of chloroethene and ethene in the headspace of the Ana* culture were determined using a gas chromatograph (GC) equipped with a flame ionization detector (FID), following the method described by Aulenta et al. [42].

### 2.7. Calculations

For PCB data calculations, co-eluting congeners and homologues were assumed to be present in equal proportions. The concentrations were expressed as ng of PCB g^−1^ of dry sediment. The PCB concentrations were calculated as the sum of the concentration of each detected PCB congener with the same chlorine number.

Volumetric reductive dechlorination rates (RD, meq/L/d) were estimated for each spike of PCE. The cumulative electron equivalents (meq) used for PCE dechlorination were calculated based on the measured amounts of dechlorination products (i.e., TCE, 1,2-cis-DCE, VC, ethene) formed during each spike, taking into account that 2 meq per mmol of chlorine are required for each removal. The maximum RDs were determined as the maximum slope value within the linear range of the curve, representing the number of molar equivalent electrons consumed during the feeding cycle. The RD was calculated from the measured dechlorination products in all the PCE spikes of the Ana* culture according to the following Equation (1):

RD (meq/L/d) = ([TCE] × 2 + [1,2 cis−DCE] × 4 +[VC] × 6 + [Eth] × 8)/V × (1/days)(1)

## 3. Results

### 3.1. PCB Contamination

PCBs were detected in the original marine sediment (138.6 ng/g dry sediment of total PCBs) used as inoculum and have been monitored in all the consortia developed.

**Figure 1:**
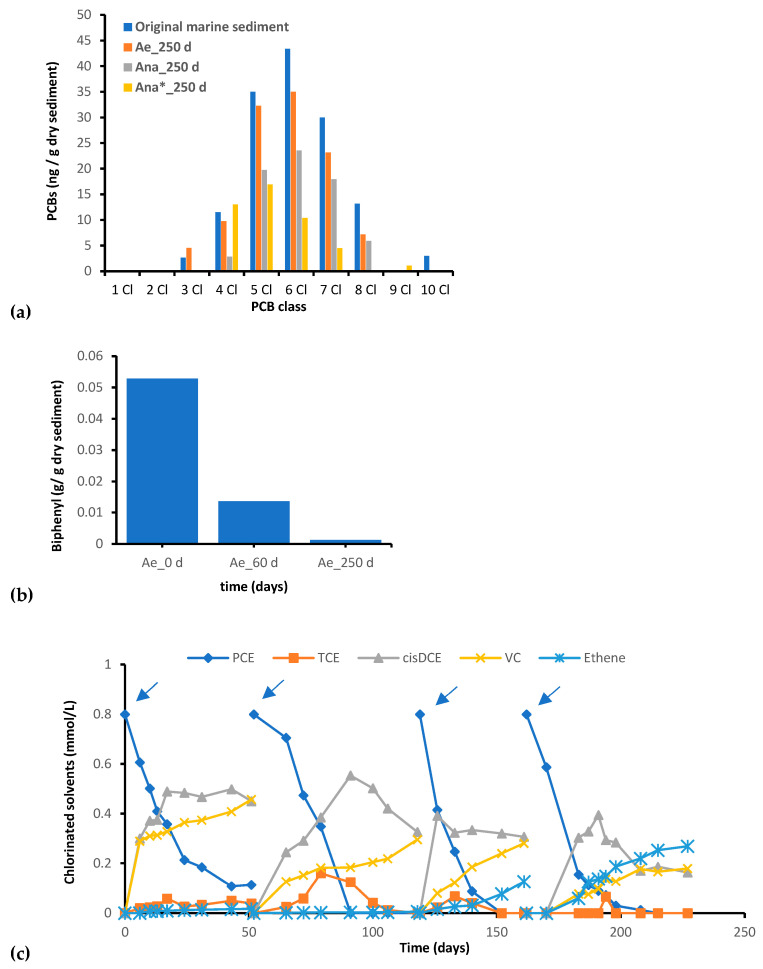
(**a**) Concentrations of PCBs and (**b**) biphenyl in consortia derived from marine sediment. PCE dechlorination pathway during three PCE/H_2_-spike events monitored during the anaerobic incubation of the Ana* culture (**c**). Blue arrows indicate the PCE spikes.

In the *Ae* culture, only a 19% cumulative reduction in the predominant PCB congeners, namely, tetra-, penta-, esa-, epta-, and octo-PCBs, was observed after 250 days of incubation (Figure 1a). By contrast, biphenyl, which was initially spiked to a concentration of 50 mg/g dry sediment to kick-start the aerobic biotransformation of PCBs, was almost completely depleted (97.6% removal) by the end of the incubation period (Figure 1b). Under anaerobic conditions, a 49% removal of total PCBs occurred after 250 days of anaerobic incubation in the *Ana* culture (Figure 1a). Notably, the most significant reductions were observed for tetra-PCBs (75% decrease) and octa-PCBs (55% decrease). Interestingly, the most extensive removal, accounting for 67%, was observed in the Ana* culture, where PCE/H_2_ was supplied to stimulate reductive dechlorination processes. Interestingly, in this culture, a substantial increase in tetra-PCBs concentration was observed, likely deriving from the reductive dechlorination of the higher chlorinated congeners. In this consortium, PCE was completely removed after each spike, accompanied by the sequential formation of dechlorination byproducts, including TCE, cis-DCE, VC, and ethene (Figure 1c). The average rate of PCE reductive dechlorination calculated on four consecutive PCE/H_2_ spike events was determined to be 0.13 + 0.02 meq/L/d. As corroborated by previous studies, the introduction of PCE enhanced reductive dechlorination processes, accelerating the reduction in heavily chlorinated PCBs and the generation of congeners with lower chlorination levels. These less chlorinated congeners are more susceptible to aerobic transformation [43].

### 3.2. Composition of the Indigenous Bacterial Community of the Multicontaminated Marine Sediment

**Figure 2:**
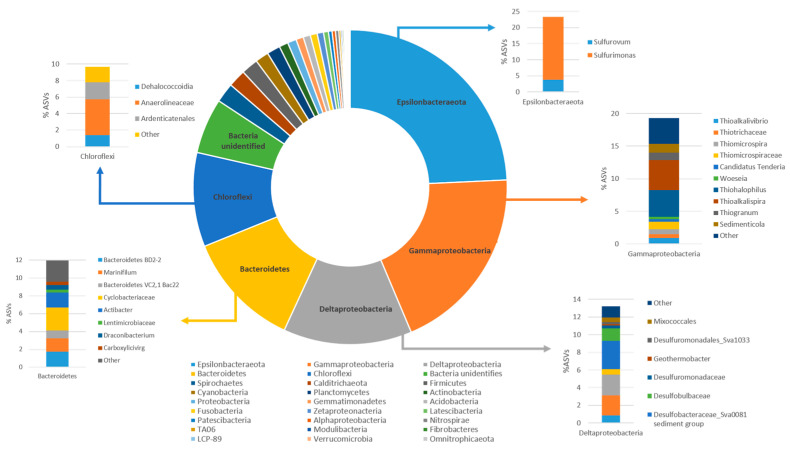
Bacterial community composition of the polluted marine sediment collected from the sampling site “Life” of the Mar Piccolo in Taranto.

The composition of the bacterial community in the marine sediment collected from the Mar Piccolo sampling station analyzed in this study was determined through 16S rRNA gene amplicon sequencing (Figure 2). The most relevant ASVs have been reported in the Supplementary Material (Table S1). The initial bacterial community displayed a Shannon index (H) of 2.3, and it was primarily composed of *Epsilonbacteraeota* (24.2%), *Gammaproteobacteria* (19.3%), *Deltaproteobacteria* (13.2%), *Bacteroidetes* (12%), and *Chloroflexi* (9.7%) as the most abundant phyla in the community.

Within the *Epsilonbacteraeota* phylum, the most prevalent genus was *Sulfurimonas* (19.5%), which comprises sulfur-oxidizing bacteria commonly found in and isolated from sulfidic habitats, especially marine sediments [14,29,44]. The *Sulfurimonas* lineage encompasses a group of sulfur-oxidizing bacteria [45] capable of using various reduced sulfur compounds, such as sulfide, elemental sulfur, thiosulfate, and sulfite, as electron donors for their growth. They are characterized by versatile energy metabolisms and adaptive abilities that are crucial for the successful colonization of various habitats. The second most abundant group was affiliated with *Gammaproteobacteria* (19.3%), primarily represented by the sulfur-oxidizing bacteria of the genera *Thiohalophilus* (4%), *Thioalkalispira* (4.6%), *Sedimenticola* (1.3%), and *Thiogranum* (1.2%). In addition, the *Deltaproteobacteria* (13.2%) identified in the contaminated marine sediment belonged to the *Desulfobacteraceae* family and included the uncultured *Sva008* group (3.2%) and *Desulfosarcina* (2%). The uncultured members of the Sva008 group have previously been studied in contaminated marine sediments and recognized as significant H_2_-scavengers in marine sediments, along with other *Desulfobacterales* [46]. The role of H_2_-oxidizing microorganisms is essential in maintaining conditions that are energetically favorable for the anaerobic degradation of organic compounds, with sulfate-reducing microorganisms being known as the main H_2_ scavengers in anoxic marine sediments. Furthermore, the presence of *Desulfosarcina* is noteworthy, as two species have been previously isolated from contaminated marine sediments as hydrocarbon-degrading sulfate-reducing bacteria [47]. Additionally, members of the phylum *Bacteroidetes* included the uncultured *Bacteroidetes* BD2-2 (1.7%) and representatives of the genera *Marinifilum* (1.5%), *Actibacter* (1.6%), and *Cyclobacteriaceae* (2.5%). These microorganisms have consistently been identified in various studies on hydrocarbon-contaminated marine sediments [48,49]. Interestingly, *Chloroflexi* members found in the marine sediment included *Anaerolineaceae* (4.4%), *Ardenticatenales* (2%), and several ASVs associated with the *Dehalococcoidia* class (1.5%). Within *Dehalococcoidia*, ASVs corresponding to uncultured bacteria were found, along with ASVs corresponding to *Dehalococcoidia*_*vadinBA26, Dehalococcoidia_MSBL5, Dehalococcoidia_GIF9_AB-539-J10_SCGC-AB-539-J10, Dehalococcoidia_FS117-23B-02,* and *Dehalococcoidia_661239.* Notably, based on 16S rRNA gene amplicon sequencing, no sequences affiliated with known *D. mccartyi* species capable of PCB reductive dechlorination were detected in the multicontaminated marine sediment sample herein investigated.

### 3.3. Dynamic Changes in the Bacterial Community during Aerobic and Anaerobic Incubation of Contaminated Marine Sediment

**Figure 3:**
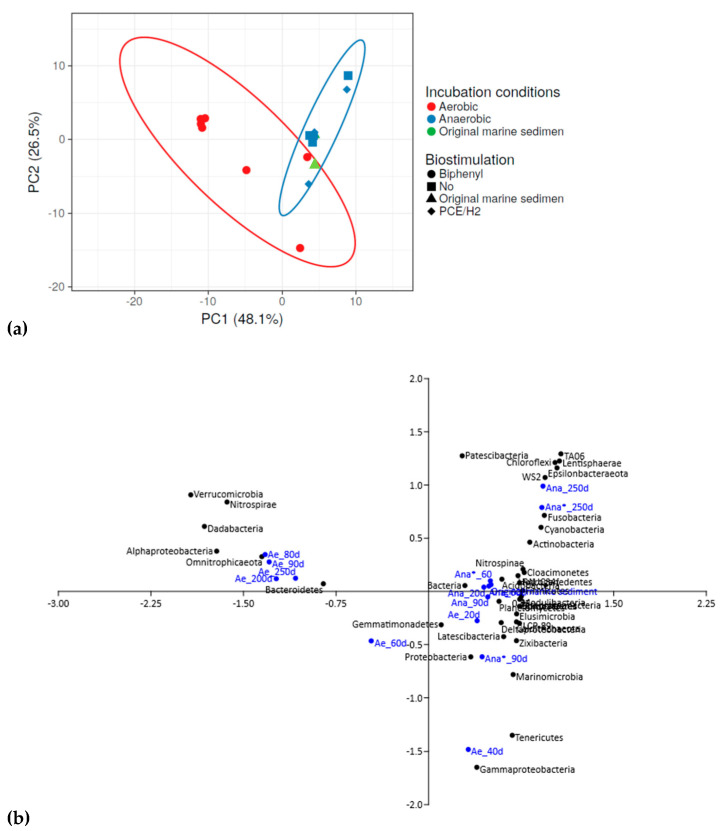
Differences in the original marine sediment’s bacterial community with different incubation conditions. Principal component analysis (PCA) (**a**) and correspondence analysis (**b**) conducted based on the inferred bacterial data and the tested biostimulation conditions. *Ae* = aerobic culture; *Ana* = anaerobic culture; Ana* = anaerobic culture with spikes of PCE/H_2_.

Incubations under both aerobic and anaerobic conditions resulted in shifts in the bacterial community structure. The overall bacterial community of the multicontaminated marine sediment exhibited a diverse response to these varying conditions (Figure 3).

In particular, as evidenced in the PCA plot, the aerobic consortium incubated with biphenyl (Figure 3a, red circles) displayed distinct changes in bacterial composition compared to the original marine sediment (Figure 3a, green triangle). Moreover, the aerobic consortium differed significantly from the anaerobic consortia developed through anaerobic incubation alone (*Ana*) (Figure 3a, blue squares) or with the addition of PCE/H_2_ (Ana*) (Figure 3a, blue rhombuses). During aerobic incubation with biphenyl (*Ae*), the bacterial community of the original marine sediment underwent dynamic shifts. Initially, this favored *Deltaproteobacteria* and *Gammaproteobacteria* (from 20 to 40 days of incubation), followed by a transition to *Alphaproteobacteria*, *Bacteroidetes*, and *Nitrospirae* (from 80 to 250 days of incubation) (Figure 3b). Conversely, under anaerobic conditions (*Ana*), the prevalent bacterial niches in the original marine sediment remained relatively stable throughout anaerobic incubations. Notably, members of the *Epsilonbacteraeota*, *Chloroflexi*, and *Gammaproteobacteria* phyla persisted during the first 90 days of anaerobic incubation (without PCE/H_2_ biostimulation), followed by the selection of *Epsilonbacteraeota* and *Chloroflexi* members after 250 days of incubation. However, a similar pattern was observed during biostimulation with PCE/H_2_ in the anaerobic consortium (Ana*) (Figure 3b). Detailed information on the bacterial community evolution of each consortium is specifically reported below.

### 3.4. Microbial Selection in the Aerobic Consortium

**Figure 4:**
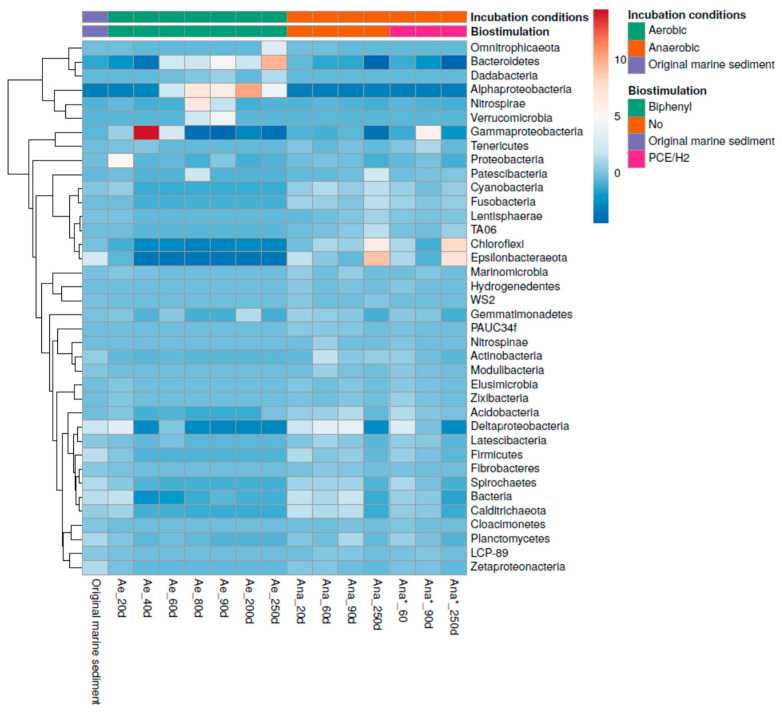
Bacterial community composition and dynamics heatmap (phylum level). The rows have been centered and subjected to Pareto scaling. Row clustering was performed using correlation distance and average linkage.

The bacterial community composition of the initial marine sediment, collected from one sample of the Mar Piccolo, underwent enrichments of specific microbial groups in the aerobic culture within just 40 days of incubation (H_0_ = 2.3; H_40 days_ = 0.48) (Figure 4).

**Figure 5:**
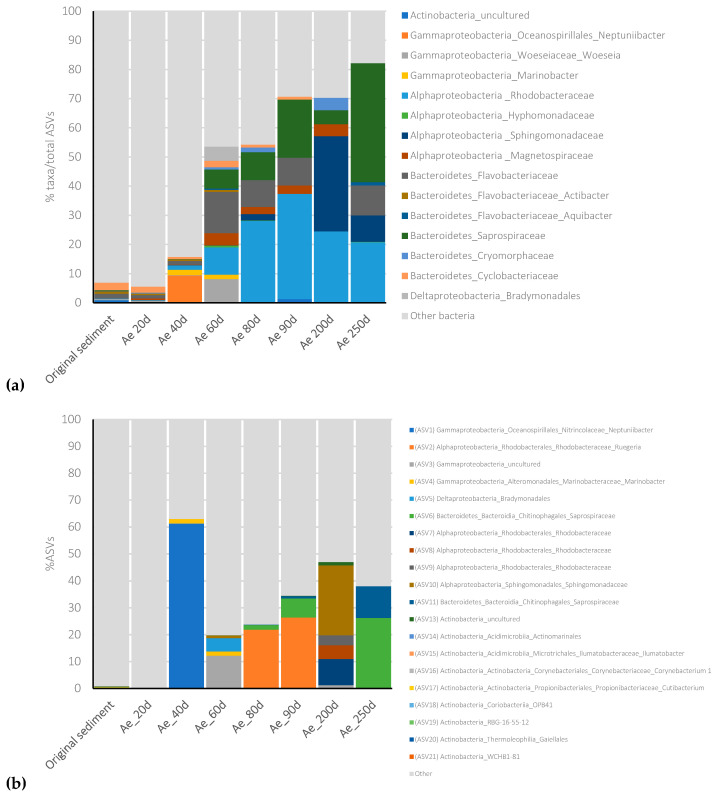

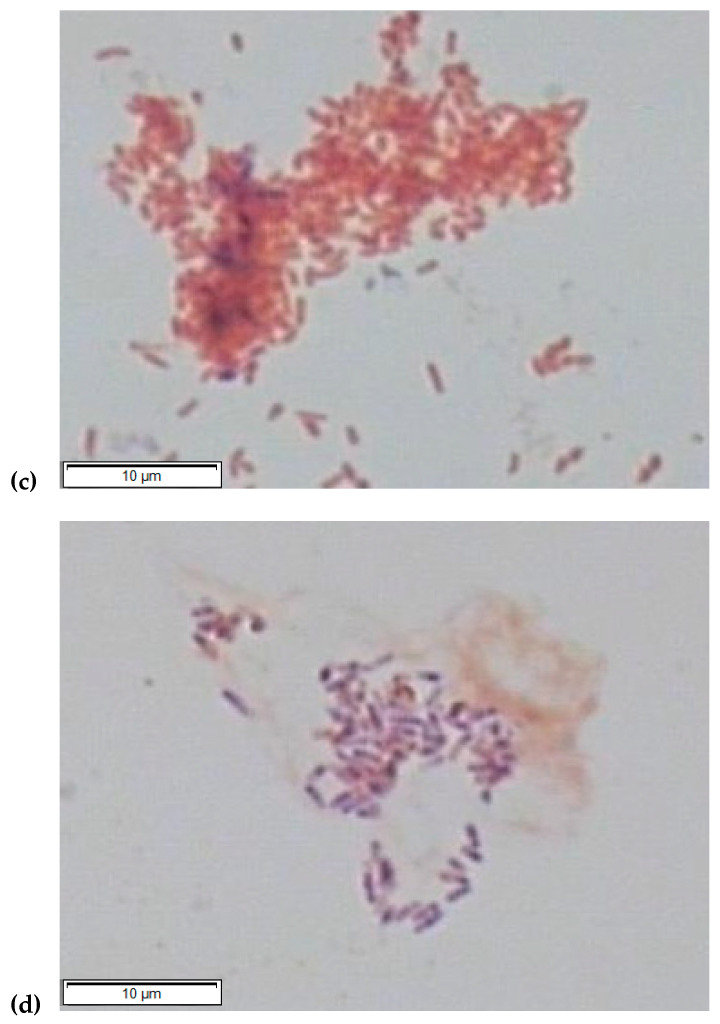
Most relevant microbial group’s dynamics (**a**) and main ASVs found (**b**) during the enrichment under aerobic incubation of the marine sediment from one sampling point of the Mar Piccolo of Taranto. *Marinobacter salinus* Gram-negative (**c**) and *Rhodococcus cerastii* Gram-positive (**d**) isolated from the aerobic consortium set up with the multicontaminated marine sediment.

*Gammaproteobacteria* dominated this stage, accounting for 91% of the community, primarily represented by members of the *Oceanospirillales* order (82%) (Figure 5a). Among them, the *Neptuniibacter* species (ASV1, Table S1, Figure 5b) constituted 62% of the entire enrichment’s bacterial community. *Oceanospirillales* members are commonly associated with marine sediments affected by oil spills, and are recognized for their involvement in aerobic hydrocarbon degradation [50,51]. Furthermore, the aerobic consortium included the obligate hydrocarbonoclastic bacterium *Marinobacter* (1.7%) (ASV4, Table S1, Figure 5b), renowned for its capacity to oxidize a variety of PAHs and aliphatic hydrocarbons. *Marinobacter* plays a pivotal role in the organic carbon cycle in contaminated environments [52]. It has been demonstrated to be involved in sulfur oxidation and the degradation of aromatic hydrocarbons. It is capable of degrading several PAHs, including naphthalene, phenanthrene, anthracene, fluoranthene, fluorine, pyrene, benz[a]anthracene, and benzo[a]pyrene, as sole carbon and energy sources, particularly in saline conditions [52,53]. Consistent with the sequencing results, we successfully isolated *Marinobacter salinus* (GeneBank accession numbers: MN108043, MN108042) by selecting on agar plates using biphenyl as the sole carbon source (Figure 5c). These findings align with additional analyses conducted on the Ae culture to elucidate the removal of PAHs. Notably, a significant reduction of 28.7% in the quantified PAHs within the marine sediment was observed after 250 days of aerobic incubation (Table S2 and Figure S1). Specifically, benzo[a]pyrene, dibenz[a,h]anthracene, benzo[ghi]perylene, and indeno[1,2,3-d]pyrene exhibited the highest levels of removal.

Similarly, after 60 days of aerobic incubation, the bacterial community exhibited a more diverse composition (H = 1.5), with *Gammaproteobacteria* at 34.3%, *Alphaproteobacteria* at 30%, *Bacteroidetes* at 25.6%, and *Deltaproteobacteria* at 8.6% as the dominant components (Figure 5a). Among *Gammaproteobacteria*, sequences included 12% of an unidentified ASV (ASV3, Table S1, Figure 5b), 8% *Woeseia* and 1.6% *Marinobacter* (Figure 5a), the latter being identified in contaminated coastal sediment and equipped with genes for complete naphthalene degradation and the oxidation of BTEX compounds [54]. Besides their hydrocarbon degradation capabilities, *Woeseiaceae* family members can also oxidize sulfite, suggesting involvement in carbon, sulfur, and nitrogen cycling in various marine sediment types [55,56]. *Alphaproteobacteria* encompassed *Rhodobacteraceae* (10%), *Hyphomonadaceae* (9.2%), *Sphingomonadaceae* (3.5%), and *Magnetospiraceae* (5%), along with other unidentified species (Figure 5a). Within *Rhodobacteraceae*, these members are frequently found in contaminated marine environments and are known to harbor a number of genes associated with aromatic degradation [56]. *Magnetospiraceae* comprises microaerophilic heterotrophs commonly found in contaminated marine sediments, exhibiting magnetotaxis and potential involvement in metal cycling [57]. *Sphingomonadaceae* and *Hyphomonadaceae* have also been identified in hydrocarbon-contaminated marine sediments and are suggested to be aerobic hydrocarbon-degrading microorganisms [58]. *Bacteroidetes* found in the *Ae* consortium included *Flavobacteriaceae* (14%, encompassing *Actibacter* and *Aquibacter* species), *Saprospiraceae* (6.5%), *Cryomorphaceae* (3.5%), and *Cyclobacteriaceae* (2.5%) (Figure 5a), which are commonly found in hydrocarbon-contaminated marine sediments [59,60]. Notably, an uncultured *Saprospiraceae* member (ASV6, Table S1, Figure 5b) increased in abundance after 90 days (7%) and 200 days of incubation (26%). Also, *Deltaproteobacteria* members of the *Bradymonadales* family were found (5% ASV5, Table S1, Figure 5b), which have been recently recognized as a predatory bacterial group widespread in marine environments [61]. Interestingly, at this sampling time (90 days), we obtained isolates of *Rhodococcus cerastii* (GeneBank accession numbers: MN108040, MN108041) (Figure 5c). *Rhodococcus cerastii*, belonging to the *Actinobacteria* phylum, specializes in oxidizing the n-alkane fraction of crude oil in marine environments. Interestingly, *Actinobacteria* members comprised only 0.9% of the total ASVs in the original sediment (ranging from ASV13 to ASV21, as listed in Table S1), and did not significantly increase over time during the aerobic incubation (Figure 5a,b). Among these ASVs, only ASV13 was associated with uncultured *Actinobacteria*, while the other ASVs were linked to members of the *Mycobacteriales, Actinomarinales, Microtrichales, Corynebacteriales, Propionibacteriales,* or *Gaiellales* orders (Table S1, Figure 5b). However, ASV13 was also detected in the aerobic enrichment after 60 and 90 days of aerobic incubation, with relative abundances of 0.14% and 1.3%, respectively.

Furthermore, we monitored the abundance of the *bphA* gene, which is responsible for encoding the bi-phenyl 2,3-dioxygenase enzyme catalyzing the initial step of the biphenyl upper pathway, throughout the aerobic cultivation in the *Ae* culture. The *bphA* gene exhibited an increase during microbial enrichment when compared to its initial abundance in the original marine sediment (≤1 × 10^3^ gene copies/g dry sediment). The highest abundance was observed after 90 days of incubation, reaching 1.7 × 10^10^ gene copies/g dry sediment (Figure S2). These findings are consistent with the presence of *bphA* redundancy in various hydrocarbon-oxidizing species [62], including the *Rhodococcus* species isolated from the consortium after 90 days of incubation. Overall, the increase in the *bphA* gene supports the idea that aerobic PCB degradation pathways were activated in the culture.

### 3.5. Microbial Community Selection in the Anaerobic Consortia

During the anaerobic incubations (both for *Ana* and Ana*), a slower selection of the bacterial community was observed compared to the aerobic enrichment culture (Figure 4). Overall, the bacterial compositions remained relatively stable during the anaerobic incubation compared to the initial marine sediment. In the initial 90 days, the dominant *phyla* in the anaerobic consortia included *Gammaproteobacteria*, *Deltaproteobacteria, Epsilonbacteraeota, Chloroflexi*, and *Bacteroidetes* (Figure 4). Particularly noteworthy was the dominance of *Thiomicrospiraceae* in the Ana* culture (36%), which significantly exceeded its representation in the *Ana* culture (2%). *Thiomicrospiraceae* are known for their autotrophic growth while oxidizing poly-metal sulfides [63]. However, after 250 days of incubation, *Epsilonbacteraeota* and *Chloroflexi* became the predominant taxa in both the *Ana* and Ana* enrichment cultures. *Epsilonbacteraeota* accounted for up to 50% and 42.5% of the *Ana* and Ana* consortia, respectively (Figure 4). Most *Epsilonbacteraeota* were closely related to a single ASV12 (Table S1) corresponding to *Sulfurimonas* species (94% sequence similarity with *Sulfurimonas autotrophica* ascertained by BLAST). This group includes sulfur-oxidizing bacteria isolated from a wide range of sulfidogenic habitats [45,64]. *Sulfurimonas* species can utilize various reduced sulfur compounds, such as sulfide, elemental sulfur, thiosulfate, and sulfite, as electron donors, highlighting their crucial role in chemoautotrophic processes. Most *Sulfurimonas* species can also grow using hydrogen as an energy source.

**Figure 6:**
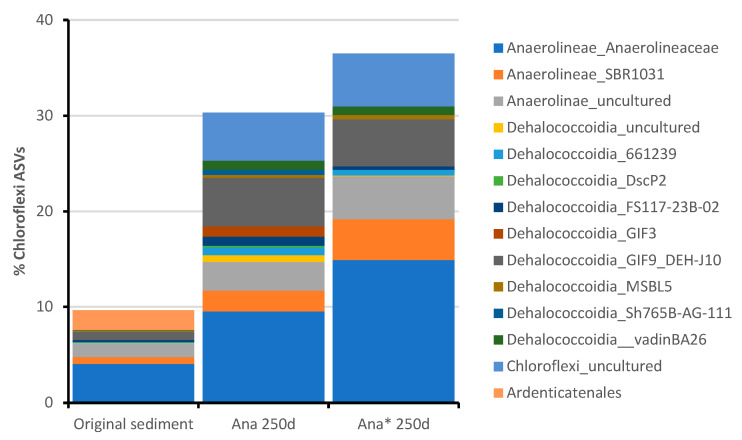
*Chloroflexi* members found in the anaerobic consortia developed after 250 days of incubation with the multicontaminated marine sediment from the Mar Piccolo of Taranto under different biostimulation conditions (*Ana* = anaerobic; Ana* = anaerobic with PCE/H_2_).

Furthermore, *Chloroflexi* dominated the anaerobic enrichments after 250 days of incubation, constituting 30.3% and 36.5% of the *Ana* and Ana* consortia, respectively (Figure 4). Within the *Chloroflexi* phylum, members of the class *Dehalococcoidia* increased from its original abundance in the marine sediment (≤1.6%) to 15.7% and 16% in the *Ana* and Ana* consortia, respectively (Figure 6).

*Dehalococcoidia* members are commonly found in anoxic environments, where they are responsible for reductive dehalogenation and potential sulfate reduction [65]. This group encompasses two formally published genera, *Dehalococcoides* and *Dehalogenimonas*, as well as the candidate genus “*Dehalobium*”, which includes obligate organohalide-respiring species [66]. *Dehalococcoidia* members are of significant interest in bioremediation efforts because they derive energy exclusively from cleaving carbon-chlorine (or bromine) bonds. These metabolic characteristics, combined with the imperative need for the cost-effective bioremediation of chlorinated contaminants, have led to extensive studies on *Dehalococcoidia* in various environmental samples, including soil, lakes, and groundwater, from which enrichment and isolation cultures have been established [67,68,69,70,71]. Notably, only a few studies have explored the distribution of *Dehalococcoidia* in marine sediments, and no *Dehalococcoidia* members have been isolated from this matrix to date. In fact, only 16S rRNA gene sequencing or single-cell sequencing has generated several incomplete *Dehalococcoidia* genomes [10,72,73,74,75]. Within the *Ana* and Ana* consortia, the most abundant *Dehalococcoidia* member found was *Dehalococcoidia_GIF9_DEH-J10*, which was already present in the original multicontaminated marine sediment used as an inoculum (Figure 6). *Dehalococcoidia_GIF9_DEH-J10* was previously detected in the marine sediments of Aarhus Bay and displayed a unique phylogenetic position within the *Dehalococcoidia* [73]. The closest cultivated strain to *DEH-J10* was *Dehalogenimonas lykanthroporepellens* strain BL-DC-9 (86% sequence identity with the 16S rRNA gene of DEH-J10). Interestingly, the *DEH-J10* genome harbored numerous genes encoding enzymes involved in beta-oxidation pathways, typically enabling the oxidation of various compounds, including fatty acids, alkanes, alkenes, aromatics, and branched-chain amino acids. Furthermore, DEH-J10 possessed genes associated with the catabolism of aromatic compounds, including benzoyl-CoA reductase and succinyl-CoA:benzylsuccinate CoA-transferase, suggesting its potential involvement in the reduction of aromatic rings, a characteristic feature of facultative anaerobes. *DEH-J10* also exhibited the capacity to oxidize substituted aromatics [76].

**Figure 7:**
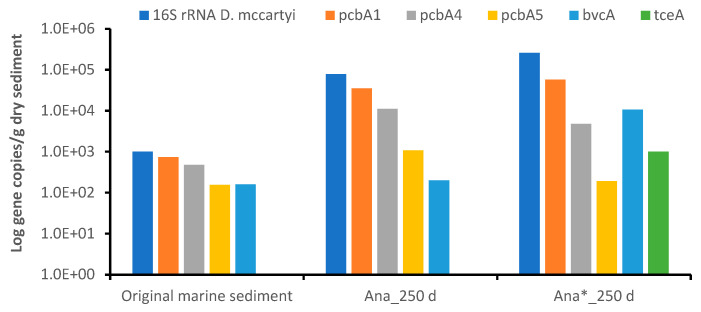
Biomarker genes involved in the PCBs’ (*pcbA1, pcbA4, pcbA*5) and PCEs’ (*tceA, bvcA, vcrA*) reductive dechlorination.

Intriguingly, ASVs affiliated with specific *D. mccartyi* strains, known for their ability to anaerobically dechlorinate PCBs and PCE, were not detected in the original marine sediment or the enrichment cultures after 250 days of incubation. However, the *D. mccartyi* 16S rRNA gene was detected in the original marine sediment, though at low concentrations (≤1 × 10^3^ gene copies/g dry sediment). Interestingly, after 250 days of anaerobic incubation, its presence increased slightly in both the *Ana* (7.84 × 10^4^ gene copies/g dry sediment) and Ana* enrichments (2.58 × 10^5^ gene copies/g dry sediment) (Figure 7). Reductive dehalogenase genes, including *pcbA1, pcbA4* and *pcbA5* genes of *D. mccartyi*, known for their involvement in PCB dechlorination [61], and the *bvcA* gene, known for the reductive dechlorination of chlorinated ethenes, were also present in the original marine sediment but at very low abundance (≤7.3 × 10^2^ gene copies/g dry sediment). Interestingly, both the *D. mccartyi* 16S rRNA and reductive dehalogenase genes increased after 250 days of incubation. In the *Ana* enrichment, *pcbA1* (3.5 × 10^4^ gene copies/g dry sediment) was the most abundant gene detected. Moreover, consistent with the observed PCB reduction and complete PCE dechlorination with Ana* enrichment, higher abundances of the screened biomarker genes were observed compared to the original marine sediment and the *Ana* enrichment. Specifically, *pcbA1* (3.5 × 10^4^ gene copies/g dry sediment) and *bvcA* (1.7 × 10^4^ gene copies/g dry sediment) were the most abundant biomarker genes identified. These findings suggest that although *Dehalococcoides* species were present in the original marine sediment at a very low abundance, their growth and dechlorination activity were stimulated by the anaerobic conditions of the bacterial enrichments and further enhanced by the addition of a direct electron donor (H_2_) and PCE, which acted as a stimulant for the reductive dechlorination process.

## 4. Discussion

In this study, we have conducted a comprehensive investigation into the bacterial communities involved in the biodegradation of polychlorinated biphenyls (PCBs) within multicontaminated marine sediment collected from one sampling point of the Mar Piccolo basin (Taranto, Italy). Our experimental design aimed to test aerobic and anaerobic conditions and identify the key microbial players responsible for PCB degradation. We utilized 16S rRNA high-throughput sequencing to investigate the dynamics of the bacterial community in the marine sediment sample. The results have revealed distinct and divergent selection patterns based on the environmental conditions applied.

In detail, the bacterial composition observed in the original marine sediment was typical of environments favoring anaerobic processes, consistent with previous studies in the same area. Indeed, a significant presence of *Epsilonbacteraeota, Gammaproteobacteria, Deltaproteobacteria, Bacteroidetes*, and *Chloroflexi* was ascertained. Among these, the prominent taxa included sulfur-oxidizing bacteria that utilize various reduced sulfur compounds as electron donors for their growth (*Sulfurimonas* species), H_2_-scavenging species (*Desulfobacteraceae*) and species typically involved in dechlorination processes, such as *Dehalococcoidia*. This composition is in line with previous studies conducted within the same basin, confirming the existence of an indigenous microbiome with the potential for hydrocarbon biodegradation, mostly for anaerobic biodegradation processes [14,18,29,30,31,32]. This initial composition served as a reference point for tracking changes in bacterial communities during incubations tested in this study. By exploring different incubation scenarios, encompassing the stimulation of aerobic dechlorination with biphenyl and various anaerobic conditions including the addition of PCE/H_2_ to prompt the reductive dechlorination process, we revealed distinct shifts in microbial communities. Incubations under both aerobic and anaerobic conditions resulted in shifts in the bacterial community structure, exhibiting a diverse response to these varying conditions. These shifts indicate the selection of specific bacteria adapted to each condition and responsive to the diverse contamination present at the site.

In the aerobic consortium, we observed significant shifts in microbial composition, with *Gammaproteobacteria* dominating early stages of incubation, followed by transitions to *Alphaproteobacteria*, *Bacteroidetes*, and *Nitrospirae* at later stages. The identification of specific bacterial species, such as *Neptuniibacter* and *Marinobacter*, known for their ability to degrade hydrocarbons, highlights their potential importance in the aerobic conversion of PCBs (polychlorinated biphenyls) in marine sediments. During the aerobic enrichment of the bacterial community of the marine sediment, the successful isolation of *Marinobacter salinus* and *Rhodococcus cerastii* has provided evidence of their presence in the native bacterial community and their potential contributions to hydrocarbon degradation within the ecosystem. Of particular significance is the likely influence of the original sediment’s PAHs on the selection of *Marinobacter salinus,* known for its heightened proficiency in degrading PAHs compared to PCBs. These findings elucidate the presence of bacterial species involved in hydrocarbon degradation beyond PCBs, which also possess functional genes relevant to PAH biodegradation. Indeed, the presence of PAHs in the initial marine sediment likely favored the proliferation of *Marinobacter salinus*, which has a greater propensity for PAH degradation than for PCBs. Typically, hydrocarbons degrade in the following order of decreasing susceptibility: n-alkanes > branched alkanes > low-molecular-weight aromatics > cyclic alkanes > PAHs > PCBs [77]. Consequently, it is plausible that *Marinobacter salinus* isolated from the contaminated marine sediment possesses a broad biotransformation capacity encompassing PAHs, PCBs, and biphenyl transformation via oxidative pathways. Nevertheless, further investigations are necessary to explore the functional roles of these species in multicontaminated marine sediments. Moreover, the isolation of *Rhodococcus cerastii* has been particularly surprising given the remarkably low occurrence of *Actinobacteria* members in both the original marine sediment and the aerobic enrichment. These findings suggest that it is possible to isolate microbial species that are initially present at low abundance in the original sample or during the enrichment culture. This success is probably attributed to the controlled and optimized conditions provided during laboratory culturing, which likely stimulate the metabolic activity of microorganisms that may be dormant or exhibit low activity in their natural environments. Indeed, microorganisms may have specific growth requirements that are not met in their natural surroundings but can be fulfilled in laboratory enrichment cultures, also allowing a gradual acclimation of microorganisms to the desired growth conditions. Over time, microorganisms that are initially present at low abundance can adapt and multiply to levels detectable through isolation techniques. Additionally, a key factor contributing to this success may be the removal of natural competition. In natural environments, microorganisms often compete for limited resources. In laboratory cultures, these competitive pressures are minimized or eliminated, allowing even low-abundance microorganisms to grow and thrive. These considerations provide evidence of the necessity to improve the combination of –omics approaches with isolation techniques to unveil the composition of the environmental microbial communities, enhance our understanding of their metabolic roles, and enable the isolation of new microbial species. In the case of complex microbiomes from contaminated sites, the more advanced stages of this approach, particularly known as “*culturomics*”, may be required. This is essential not only for determining microbial composition, but also for obtaining microbial isolates and cultures, by testing a wide array of conditions, which can be applied in bioremediation strategies on the site.

In the anaerobic consortia, bacterial communities exhibited slower changes compared to the aerobic culture, with relatively stable compositions during the initial 90 days of incubation. Notably, *Epsilonbacteraeota* and *Chloroflexi* became the predominant taxa after 250 days of incubation in both the *Ana* and Ana* consortia. The presence of *Sulfurimonas* species within *Epsilonbacteraeota* indicates their potential role as sulfur-oxidizing bacteria in chemoautotrophic processes, while the presence of members of the class *Dehalococcoidia* in *Chloroflexi* suggests the potential for the reductive dechlorination of PCBs. Genes involved in reductive dehalogenation, such as *rdhA* genes, were detected in the anaerobic consortia, further supporting the hypothesis that certain microorganisms in the culture were capable of PCB dechlorination under anaerobic conditions. Additionally, the presence of sulfate-reducing microorganisms suggests the occurrence of sulfur cycling processes in the anaerobic consortia. These findings highlight the need for further investigations, including metagenomic analysis and single-cell genome sequencing, to clarify the identity of specific microorganisms, particularly those affiliated with the *Dehalococcoidia* found in the anaerobic consortia. Understanding their roles in anaerobic PCB dechlorination processes will contribute to a more comprehensive understanding of PCB biotransformation in complex environments.

## 5. Conclusions

Our study has provided valuable insights into the potential microbial factors and processes involved in PCB degradation under various incubation conditions, by analyzing the marine sediment collected from one sampling point of the Mar Piccolo basin in Italy. The observed shifts in bacterial communities have shed light on the adaptation of microorganisms to the tested conditions and their potential roles in PCB transformation processes, but also the biodegradation potentialities of other co-contaminant hydrocarbons such as PHAs. From the indigenous microbial community in the highly contaminated sediment, we successfully obtained three distinct enrichment cultures and two isolates. One bacterial consortium showed the potential to reduce the presence of PCBs and PAHs under aerobic conditions (consortium *Ae*), and allowed us to extrapolate two bacterial isolates (*Marinobacter salinus* and *Rhodococcus cerastii*) known to play a role in the biodegradation of hydrocarbons. In addition, two anaerobic cultures were obtained, able to perform PCB biodegradation via reductive dechlorination (consortium *Ana*) and to perform the complete reductive dechlorination of chlorinated ethenes (consortium Ana*). The establishment of consortia with biodegradation and biotransformation capabilities expands the toolkit available for effectively implementing bioremediation strategies, thus enhancing the comprehensive nature-based restoration approach. Hence, in our current study, we conducted microbial characterization through 16S rRNA gene amplicon high-throughput sequencing to assess bacterial selection. However, to gain a deeper understanding of the metabolic interactions within the developed consortia, we plan to undertake further investigations, including a comprehensive metagenomic study encompassing the entire microbial community, spanning both *Bacteria* and *Archaea* domains.

## Figures and Tables

**Table 1:** Selected Ion Monitoring MS method used for PCBs determination.

Cl	*m*/*z*	Time Range (min)
- *	154.1	5–10
1	188.1–190.1	10–13
2	222.0–224.0	12.2–18
3	256.0–258.0	15–23
4	291.9–293.9	18–29
5	325.9–327.9	21–32.5
6	359.9–361.9	24.5–34.5
7	393.9–397.8	29–35.5
8	429.8–431.8	32–36.5
9	463.8–465.8	34.5–37
10	497.7–499.7	36–41.5

* biphenyl.

## Data Availability

Sequencing data produced in this study have been deposited in the DDBJ/ENA/GenBank under the BioPro-ject PRJNA843975.

## Supplementary Materials

The following supporting information can be downloaded at: https://www.mdpi.com/article/10.3390/microorganisms11112782/s1, Figure S1. PAHs removal from the Ae microbial consortium during the aerobic incubation of the contaminated marine sediment from the Mar Piccolo of Taranto; Figure S2. bphA gene quantified in the Ae consortium during the aerobic incubation of the contaminated marine sediment from the Mar Piccolo of Taranto; Table S1. 16S rDNA sequence of the most relevant ASVs observed in the enrichment cultures (deposited in DDBJ/ENA/GenBank under the BioProject PRJNA843975); Table S2. PAH concentrations obtained in the original marine sediment and in the aerobic consortium after 250 days of incubation (Ae_250d).
