# Supplementary material for: Enrichment of Aerobic and Anaerobic Hydrocarbon-Degrading Bacteria from Multicontaminated Marine Sediment in Mar Piccolo Site (Taranto, Italy)
Source: Microorganisms. 2023 Nov 16;11(11):2782. doi: 10.3390/microorganisms11112782 (PMC10673493; doi:10.3390/microorganisms11112782)
Supplement: Supplementary file 1 [file microorganisms-11-02782-s001.zip › microorganisms-2671472-supplementary.pdf]

microorganisms

MDPI

Supplementary Material

Enrichment of Aerobic and Anaerobic Hydrocarbon-Degrading Bacteria from Multicontaminated Marine Sediment in Mar Piccolo Site (Taranto, Italy)

Bruna Matturro [1,2,*], Maria Letizia Di Franca [1], Barbara Tonanzi [1,2], Carolina Cruz Viggi [1], Federico Aulenta [1,2], Magda Di Leo [1], Santina Giandomenico [1] and Simona Rossetti [1]

[1] Water Research Institute (IRSA), National Research Council (CNR), 00010 Montelibretti, Italy; federico.aulenta@irsa.cnr.it (F.A.); simona.rossetti@irsa.cnr.it (S.R.)

[2] National Biodiversity Future Center, 90133 Palermo, Italy

* Correspondence: bruna.matturro@irsa.cnr.it

Citation: Matturro, B.; Di Franca, M.L.; Tonanzi, B.; Cruz Viggi, C.; Aulenta, F.; Di Leo, M.; Giandomenico, S.; Rossetti, S. Enrichment of Aerobic and Anaerobic Hydrocarbon-Degrading Bacteria from Multicontaminated Marine Sediment in Mar Piccolo Site (Taranto, Italy). *Microorganisms* **2023**, *11*, 2782. https://doi.org/10.3390/microorganisms11112782

Academic Editors: Johannes F. Imhoff and Zhiyong Li

Received: 20 October 2023

Revised: 7 November 2023

Accepted: 14 November 2023

Published: 16 November 2023

Copyright: © 2023 by the authors. Licensee MDPI, Basel, Switzerland. This article is an open access article distributed under the terms and conditions of the Creative Commons Attribution (CC BY) license (https://creativecommons.org/licenses/by/4.0/).

1. *PAH determination*

As for PAHs determination, after sampling soil was air dried at room temperature for 48 h on a hexane-rinsed aluminium foil and finely ground in an agate mortar. The extraction was performed using a Microwave-assisted Extraction system (Ethos X, Milestone). Five grams of dried samples were accurately weighed into disposable glass vials of 100 mL. This was followed by the addition of a 25-mL 1:1 acetone/hexane solvent mixture. The extraction was performed for 15 min at 110 °C and 6–10 bars; then, extracts were cooled to room temperature and treated with Na2SO4 cartridges. Finally, extracts were concentrated by evaporation under a gentle nitrogen stream to a final volume of 5 mL. The extract (1 µL) was then injected (in pulsed split-less mode) into a GC–MS (Perkin Elmer Clarus 680/600; column: HP-5 MS (Agilent) 30 m, ID 0.25 mm, 0.25 mm film thickness; carrier gas: helium 1 mL/min; Inj Temp: 310 °C; Interface Temp: 280 °C; Oven Temp Program: 50 °C for 0.0 min, then 20 °C/min to 100 °C, then 5 °C/min to 300 °C, then 300 °C for 2.5 min. MS method: Selected Ion Monitoring (m/z: 78, 91, 128, 152, 154, 166, 178, 202, 228, 252, 276, 278). Quantification of PHAs was performed by means of external standards containing the 16 priority PAH Pollutants (PAH Calibration Mix, TraceCERT®, Merck).

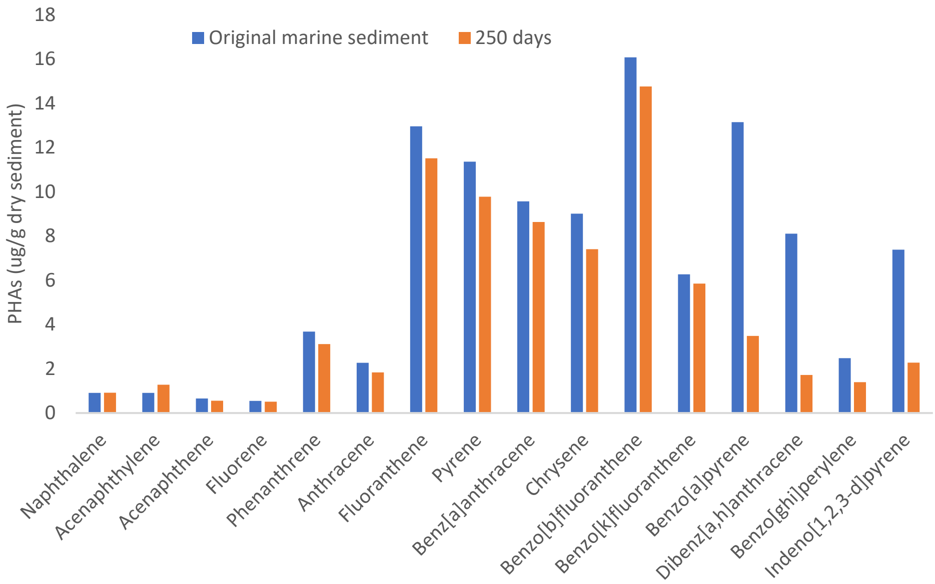

Figure S1. PAHs removal in the Ae microbial consortium during the aerobic incubation of the contaminated marine sediment from the Mar Piccolo of Taranto.

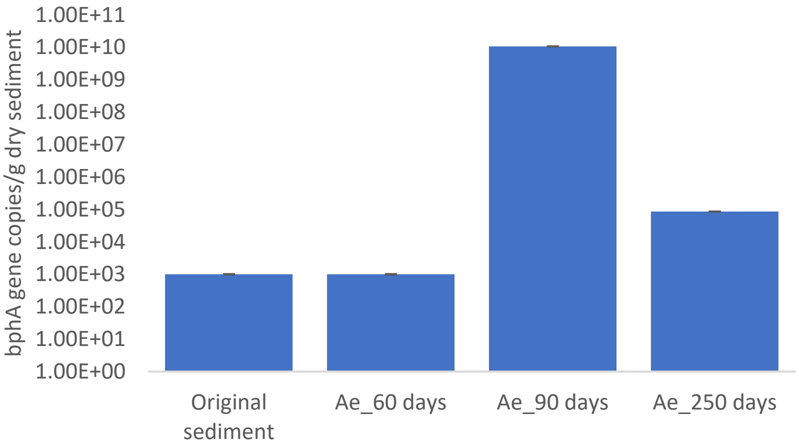

Figure S2. *bphA* gene quantified in the Ae consortium during the aerobic incubation of the contaminated marine sediment from the Mar Piccolo of Taranto.

Table S1. 16S rDNA sequence of the most relevant ASVs observed in the enrichment cultures (deposited in DDBJ/ENA/GenBank under the BioProject PRJNA843975).

ASV ID	DNA Sequence Identifier	Taxonomy
ASV1	cecf47014e6a5dc2e866107ad8e96cbf	Gammaproteobacteria_Oceanospirillales_Nitrincolaceae_Neptuniibacter
ASV2	ec3c872ac1f8cd010f4fa9d252032690	Alphaproteobacteria_Rhodobacterales_Rhodobacteraceae_Ruegeria
ASV3	10b89078322301150e3ac51ecc1094c2	Gammaproteobacteria_pltb-vmat-80
ASV4	b2d02dcd71a08b5c472160873306a270	Gammaproteobacteria_Alteromonadales_Marinobacteraceae_Marinobacter
ASV5	40fe9c3cef236d812aedf7783e157fd9	Deltaproteobacteria_Bradymonadales
ASV6	5da17d5b4a800e53977ce8630c035cc1	Bacteroidetes_Bacteroidia_Chitinophagales_Saprospiraceae
ASV7	a8780a1ef91d3fc5536c1a05ef672b62	Alphaproteobacteria_Rhodobacterales_Rhodobacteraceae

ASV8	f792eeb9813e01901702879c28209e9d	Alphaproteobacteria_Rhodobacterales_Rhodobacteraceae
ASV9	9b29a67b6a67ae7ab6f13df2ae2a2a3a	Alphaproteobacteria_Rhodobacterales_Rhodobacteraceae
ASV10	58bfe8b92abb088494a7851006847e59	Alphaproteobacteria_Sphingomonadales_Sphingomonadaceae
ASV11	6f54955399f4c1b006b7e1e21aae3fa6	Bacteroidetes_Bacteroidia_Chitinophagales_Saprospiraceae
ASV12	12eda78fccac9f432a226f7d38e3150c	Epsilonbacteraeota_Campylobacteria_Campylobacterales_Thiovulaceae_Sulfurimonas
ASV13	ae8be099f38303539ca6a01d2b5412f5	Actinobacteria_uncultured
ASV14	1d5140252e0ef3ec8512549a8041cf09	Actinobacteria_Acidimicrobiia_Actinomarinales
ASV15	a7ac4a65e33612728f0f08f036a9a8d3	Actinobacteria_Acidimicrobiia_Microtrichales_Ilumatobacteraceae_Ilumatobacter
ASV16	04ae6d4e93605dc0fd87868b6069bbbf	Actinobacteria_Actinobacteria_Corynebacteriales_Corynebacteriaceae_Corynebacterium 1
ASV17	2c38d9d5742202da694d4721608fd637	Actinobacteria_Actinobacteria_Propionibacteriales_Propionibacteriaceae_Cutibacterium
ASV18	e3ebba3fc0a45df12b84849a58f6e20e	Actinobacteria_Coriobacteriia_OPB41
ASV19	b2c92fa4fe28862db2bd8913e695a2a8	Actinobacteria_RBG-16-55-12
ASV20	2000c1fc5eda187e4f6c4bd058a86c5f	Actinobacteria_Thermoleophilia_Gaiellales
ASV21	3545b09c3674713988f8ea609271f5a7	Actinobacteria_WCHB1-81

Table S2. PAH concentrations obtained in the original marine sediment and in the aerobic consortium after 250 days of incubation (Ae_250d).

	**Original marine sediment (µg/g dry sediment)**	**Ae_250 d (µg/g dry sediment)**
**Naphthalene**	0.907	0.916
**Acenaphthylene**	0.906	1.277
**Acenaphthene**	0.657	0.556
**Fluorene**	0.550	0.511
**Phenanthrene**	3.675	3.112
**Anthracene**	2.266	1.837
**Fluoranthene**	12.954	11.506
**Pyrene**	11.360	9.776
**Benz[a]anthracene**	9.562	8.629
**Chrysene**	9.006	7.402
**Benzo[b]fluoranthene**	16.071	14.758
**Benzo[k]fluoranthene**	6.270	5.848
**Benzo[a]pyrene**	13.143	3.486
**Dibenz[a,h]anthracene**	8.103	1.719
**Benzo[ghi]perylene**	2.478	1.393
**Indeno[1,2,3-d]pyrene**	7.378	2.278
